# Corticosteroids in lung cancer

**DOI:** 10.3389/fdsfr.2025.1528468

**Published:** 2025-04-24

**Authors:** Vesna Ćeriman Krstić, Milija Gajić, Leonida Djukanović, Dragana Jovanović

**Affiliations:** ^1^ Faculty of Medicine, University of Belgrade, Belgrade, Serbia; ^2^ Clinic for Pulmonology, University Clinical Center of Serbia, Belgrade, Serbia; ^3^ Municipal Institute for Lung Diseases and TB, Belgrade, Serbia; ^4^ Internal Medicine Clinic, “Akta Medica”, Belgrade, Serbia

**Keywords:** corticosteroids, lung cancer, immunotherapy, pain, cachexia, dyspnea, nausea and vomiting, brain metastases

## Abstract

Despite significant advances in lung cancer treatment, patients with this disease still present with multiple symptoms that are very hard to control. Corticosteroids are widely used in patients with lung cancer, but without clear evidence for their efficacy. Thus, corticosteroids have been used for the treatment of conditions arising due to the tumor itself, adverse effects of the applied specific therapy and symptom palliation. In this review we are going to summarize clinical indications for corticosteroid use in patients with lung cancer: malignant airway obstruction, superior vena cava syndrome, brain metastases, treatment-related adverse events, anorexia and cachexia, fatigue, dyspnea, nausea and vomiting, spinal cord compression, and pain.

## 1 Introduction

Despite significant advances in the treatment of lung cancer, it still represents a major cause of death among cancer patients, with multiple symptoms that are very hard to control, especially at the end of life when only palliative care could be offered (Yao et al., 2023). The data from “GLOBOCAN” showed that lung cancer was the most commonly diagnosed cancer in 2022, and also it was the leading cause of cancer death among other cancers (Bray et al., 2024).

In the treatment of some conditions and symptoms, corticosteroids are widely used. But there is little evidence for their efficacy (Yao et al., 2023). Also, in the era of immunotherapy and molecular therapy, corticosteroids are the backbone in the treatment of adverse events (Nauck et al., 2004).

Some studies investigated the use of corticosteroids in cancer patients. Thus, Nauck et al. (Yennurajalingam et al., 2011) conducted a large multicenter study in palliative care units. They found that corticosteroids were administered in 33% of patients, and also they found that younger patients were treated more often with corticosteroids (Nauck et al., 2004).

In another study, it was reported that corticosteroids were administered in about 25% of patients in an outpatient clinic Yennurajalingam et al. (2011) and Gannon and McNamara (2002), found in their retrospective study that corticosteroids were prescribed in 51% of patients at the end of life, and it was continued until death in 53% of patients.

Also, it was investigated which of the corticosteroid drugs could be the best option and in which dose. Hanks et al. (1983), Lossignol (2016) evaluated the efficacy of prednisolone versus dexamethasone in over 300 patients with advanced cancer. There was no difference in response between the two drugs (Hanks et al., 1983; Lossignol, 2016).

Vecht et al. (Lossignol, 2016; Vecht et al., 1989) investigated which dose of dexamethasone, 10 mg versus 100 mg, could be more effective in pain relief in patients with spinal cord compression. No significant difference was found between these two groups of patients (Lossignol, 2016; Vecht et al., 1989).

Liu et al. (2021) conducted a study in which they identified the five most frequent symptoms within chemotherapy in patients with non-small cell lung cancer (NSCLC). The study included 127 patients with NSCLC who were treated with platinum based therapy (Liu et al., 2021). It included fatigue, insomnia, cough and sputum, appetite loss, and hypodipsia (Liu et al., 2021). A significant difference was found in fatigue, insomnia, cough and sputum, and appetite loss in a group of patients who received dexamethasone compared to the other group in which patients received a placebo (Liu et al., 2021). The symptoms were improved, and also the quality of life (Liu et al., 2021).

Corticosteroids have been used for the treatment of multiple conditions in lung cancer patients, such as conditions arisen due to the tumor itself, adverse effects of the applied specific therapy, and symptom palliation.

As it was already mentioned, corticosteroids are widely used in multiple conditions in lung cancer patients. However, there are also side effects of their use. The large literature review included 32 articles in order to investigate the most commonly reported side effects of corticosteroids after long-time use (Rice et al., 2017). The results showed that the most commonly found are cataract (1%–3%), nausea/vomiting/other gastrointestinal conditions (1%–5%), sleep disturbance, bone fracture (21%–30%) or osteoporosis, cardiac conditions (including myocardial infarction), type 2 diabetes mellitus and hyperglycemia, and hypertension (>30%) (Rice et al., 2017). Thus, we should be very careful when prescribing corticosteroids, especially in patients with long-time survival expectations.

## 2 Conditions arisen due to tumor

### 2.1 Malignant central airway obstruction

Malignant central airway obstruction is defined as limited airway flow in the trachea, main bronchi, and bronchus intermedius (Powers and Schwalk, 2023). It can be found in patients with primary lung tumors but also in patients with metastatic disease (Powers and Schwalk, 2023).

This condition can be presented as mild dyspnea but also with stridor and severe respiratory compromise (Jovanovic et al., 2024).

Surgical treatment is not an option in the majority of cases due to advanced disease (Powers and Schwalk, 2023). Different bronchoscopic interventional procedures are required in the treatment of central airway obstruction (Powers and Schwalk, 2023).

There is also some data about the use of high-dose of corticosteroids in this setting (Lin et al., 2012; Elsayem and Bruera, 2007). It is assumed that high doses of corticosteroids can reduce tumor related airway edema or secretions (Lin et al., 2012).

### 2.2 Superior vena cava syndrome

Superior vena cava (SVC) syndrome can occur due to complete or partial obstruction of the superior vena cava (Jovanovic et al., 2024). The most common symptoms and signs of SVC obstruction are face or neck swelling, upper extremity swelling, and dyspnea (Jovanovic et al., 2024; Patriarcheas et al., 2022).

The therapeutic approach of SVC syndrome implies alleviation of symptoms related to SVC obstruction and treating the underlying disease (Patriarcheas et al., 2022; Wilson et al., 2007). General measures imply elevation of the patient’s head with the goal of decreasing head and neck edema and hydrostatic pressure, administration of corticosteroids and diuretics (recommended in the literature - without clear evidence of efficacy) (Patriarcheas et al., 2022; Wilson et al., 2007). Diuretics can reduce venous return to the heart and relieve the increased pressure (Patriarcheas et al., 2022; Wilson et al., 2007). But it should be administered with caution because the problem is not the fluid overload but the obstruction of blood flow through the SVC. Corticosteroids are usually used in order to reduce the development and extension of edema and in that way to decrease extrinsic pressure on the SVC (Patriarcheas et al., 2022; Shah et al., 2023; Wright et al., 2023; Chow et al., 2024). However, there is little data about their use in this setting, and also there is no consensus about the optimal dose and duration (Wright et al., 2023).

In Table 1 are shown recommended doses of dexamethasone in some clinical situations.

**TABLE 1:** Recommended dose of dexamethasone in some clinical indications.

Clinical indication	Recommended dose, *mg*
Raised intracranial pressure	8–16 mg dexamethasone daily
Spinal cord compression	16–32 mg dexamethasone daily (8–16 mg b.i.d.)
Superior vena cava obstruction	16–24 mg dexamethasone daily (8 mg b.i.d or t.i.d.)
Bowel obstruction	8–16 mg dexamethasone daily
Anorexia	4 mg dexamethasone; 10–20 mg prednisolone
Nausea and vomiting	4–8 mg dexamethasone
Bone and neuropathic pain	4–8 mg dexamethasone

### 2.3 Brain metastases

Brain metastasis is a frequent neurologic complication of lung cancer detected in a maximum of 40% of patients. The most common symptoms are headache, which occurs due to increased cerebral edema with increased intracranial pressure and meningeal irritation secondary to tumor cell infiltration, and focal neurologic deficits, which can cause cognitive disorder, and/or motor and sensory function loss. Increased intracranial pressure is caused by single or multiple brain metastases, regardless of the number of them. Early recognition and treatment are essential to prevent neurologic worsening leading to seizures, deterioration of mental status, and coma.

Corticosteroids are routinely used to reduce cerebral edema and alleviate symptoms due to high intracranial pressure or focal neurologic symptoms (Ly and Wen, 2017; Jessurun et al., 2019; Chang et al., 2019).

Use of corticosteroids has particularly shown good efficacy in the treatment of vasogenic edema in patients with larger and multiple brain metastases (higher numbers of brain metastases), even regardless of primary tumor type and dosing of the drug (Schroeder et al., 2019).

Dexamethasone is the treatment of choice due to the least mineralocorticoid effect and being less likely to foster infection or cognitive dysfunction compared with other corticosteroids. The dexamethasone dose used in routine clinical practice is most often 4 mg given intravenously or orally every 6 h after an initial dose of 10 mg, thus improving symptoms in 70%–80% of patients within 48 h of the introduction of therapy (Jessurun et al., 2019; Palombi et al., 2018; Drappatz et al., 2007).

Evidence on the safety and efficacy of different dexamethasone doses in malignant brain tumor patients is scarce and conflicting. Most studies reported a dose of 16 mg, commonly in doses of 4 mg given 4 times a day as well (Jessurun et al., 2019).

Best available evidence suggests that higher doses of dexamethasone may induce more adverse events, but may not necessarily result in better clinical condition. Some studies suggest that higher dexamethasone doses are associated with shorter survival in the palliative setting. Several studies reported that dexamethasone ≤8 mg/day was associated with significantly longer survival compared with >8 mg/day in BM patients receiving WBRT (Jessurun et al., 2019; Priestman et al., 1996; Tang et al., 2008).

Thus, lower doses of dexamethasone may produce similar clinical benefit at the same time with fewer adverse events when compared to higher doses (Schroeder et al., 2019).

While symptomatic improvement is usually seen within 24–72 h, the use of dexamethasone is associated with some serious adverse events including muscular weakness, hyperglycemia and diabetes, cushingoid symptoms, increased rate of opportunistic infection, mental disorders, and gastrointestinal ulceration and bleeding (Drappatz et al., 2007; Kural et al., 2018; Kaur et al., 2023; Sturdza et al., 2008).

Worse survival with higher doses as observed in few studies could be explained by antiproliferative properties of dexamethasone that may prevent radiotherapy and chemotherapy-induced genotoxic stress. Individual response to dexamethasone varies most probably due to polymorphisms of the glucocorticoid receptor gene (Ryan et al., 2012; Huizenga et al., 1998).

Moreover, the drug clearance could be disturbed by anticonvulsants that may induce or inhibit cytochrome P450 liver enzymes (Ly and Wen, 2017).

It should be noted that individual variation in plasma free fraction could cause variation in (severity of) adverse events as well (Ryan et al., 2012).

Also, as the biologic half-life of dexamethasone is 34–54 h, doses may not have to be applied four times a day (Jessurun et al., 2019).

It should be underlined that the frequency of corticosteroid complications also depends on the duration of treatment, with >3 weeks increasing the risk, and on the cumulative dose of dexamethasone as well (Ryan et al., 2012).

The interactions between dexamethasone and immunotherapies represent a great challenge that should be studied more in patients with brain metastases. In many ways, corticosteroids and immunotherapies have directly antagonistic effects (Maxwell et al., 2018; Garant et al., 2017; Giles et al., 2018; Goodman et al., 2023), but the existing data on the effect of systemic corticosteroids on immunotherapy efficacy remain somewhat conflicted and unclear.

The well-known mechanisms of corticosteroids immunosuppression relevant for immunotherapy efficacy include impairment of IL-2 mediated effector T cell activation and increase of Treg cells as well as promoting macrophage polarization and altering the microbiome. So, in cancer, corticosteroids may affect the release of tumor antigens, lymphocyte function, and immune-mediated tumor cell destruction, thus counteracting each other (Table 2) (Giles et al., 2018). The ESMO Clinical Practice Guidelines provide symptom-specific approach suggestions as well as potential steroid-sparing treatments (Crawford et al., 2021) such as Corticorelin Acetate (Recht et al., 2013).

**TABLE 2:** Antagonistic effects of immunotherapy and corticosteroids on the immune system.

Immunotherapy Upregulation of tumor response	Corticosteroids immunosuppression
Increased activation and proliferation of tumor- reactive CD8^+^ T cells	Reduced memory CD8^+^ T cells
Increased immune cells	Decreased monocytes, macrophages, lymphocytes, eosinophils, and basophils
Increased pro- inflammatory cytokines (IFN-gamma, TNF, IL-17)	Suppression of pro- inflammatory cytokines (IL-2, IL-6, TNF- alpha) and prostaglandins
Reduced regulatory T cells • Decreased anti- inflammatory cytokines (TGF- beta, IL-10)	Increased regulatory T cells • Increased anti- inflammatory cytokines (TGF- beta, IL-10)

There is some evidence indicating that patients with brain metastases treated with immunotherapy may have worsened survival if they are concurrently receiving corticosteroids for brain metastases (Margolin et al., 2012; Kotecha et al., 2019).

Moreover, there is evidence suggesting that corticosteroids may have some metastasis-inducing properties (Obradović et al., 2019). Namely, the results of the study conducted by Obradovic et al. suggested increased glucocorticoid receptor activity in distant metastases in patients with breast cancer; thus, caution is needed when use glucocorticoids to treat patients for cancer-related complications (Obradović et al., 2019).

## 3 Corticosteroids in the management of treatment-related adverse events

Immune check point inhibitors made a revolution in the treatment of many cancers, including lung cancer. Adverse events can occur at any time during the treatment, even after stopping the treatment, but the majority of them occur at 12–16 weeks from immunotherapy initiation (Gould Rothberg et al., 2022). The combination of two immunotherapy agents precipitates immune related adverse events (irAE) earlier than monotherapy (Gould Rothberg et al., 2022). Some studies even suggest that patients who had mild to moderate irAE had better outcomes compared to patients who did not have irAE (Gould Rothberg et al., 2022). The immune-related adverse events are mainly treated with high-dose corticosteroids- 1–2 mg/kg daily (Gould Rothberg et al., 2022). Table 3.

**TABLE 3:** Selected irAE treated with corticosteroids.

a) Diarrhea/colitis			
Differential diagnosis	Grade 1 (mild)	Grade 2 (moderate)	Grade 3-4 (severe)
Infection IBD Primary cancer of the colon	Symptomatic treatment; Consider budesonide 9 mg/day; Continue IO	Delay IO; Methylprednisolone iv 0.5–1 mg/kg/day (or oral equivalent); Consider gastroenterology consultation and colonoscopy; If improve to grade ≤1 reduce dose for at least 4 weeks	Stop IO; Methylprednisolone iv 1–2 mg/kg/day; If improve to grade ≤1 reduce dose for at least 4 weeks If no improvement within 48–72 h consider 2nd line immunosuppression (infliximab)

b) Hepatitis			
Differential diagnosis	Grade 1 (mild)	Grade 2 (moderate)	Grade 3–4 (severe)
Infection Other concomitant drug reaction Alcohol intake Biliary disease	Continue IO; Repeat LFTs within 1 week	Delay IO; Repeat LFTs every 3–5 days; Methylprednisolone iv 0.5–1 mg/kg/day (or oral equivalent); If improve to mild or baseline, reduce the dose of steroids for at least 4 weeks	Stop IO; Increase the frequency of LFTs to 1–2 days; Methylprednisolone iv 1–2 mg/kg/day; Consult gastroenterologist; If no improvement in symptoms within 48–72 h, consider 2nd line immunosuppression (infliximab)

c) Dermatological AEs			
Differential diagnosis	Grade 1 (mild)	Grade 2 (moderate)	Grade 3–4 (severe)
Inflammatory or other non- inflammatory disease	Continue IO; Supportive therapy emollients, low-potency topical steroids, antihistamines	Continue IO; Topical steroids of moderate-high potency; If persistent, despite optimized topical treatment, consider methylprednisolone 0.5–1 mg/kg/day (or oral equivalent); If it improves slightly or resolves, reduce the dose of steroids for at least 4 weeks; Consider dermatological evaluation and skin biopsy	Delay IO; Methylprednisolone IV 1–2 mg/kg/day (or oral equivalent)If it improves to mild or resolves, reduce the dose of steroids for at least 4 weeks; Consider skin biopsy

d) Pneumonitis			
Differential diagnosis	Grade 1 (mild)	Grade 2 (moderate)	Grade 3–4 (severe)
Pneumonia Tuberculosis Fungal infection COVID-19 infection	Delay IO; Monitor symptoms; Repeat chest X-ray in 2–4 weeks	Delay IO; Monitor symptoms closely, consider hospitalization; Re-image every 1–3 days; Pulmonology and infectious disease consultations, consider bronchoscopy; Methylprednisolone iv 1–2 mg/kg/day (or oral equivalent) If symptoms improve, reduce the dose of steroids for at least 4 weeks	Stop IO; Methylprednisolone iv 2–4 mg/kg/day, discontinue steroids for a period of at least 6 weeks; If no improvement in symptoms within 48–72 h, consider 2nd line immunosuppression (infliximab, mycophenolate mofetil, IVIG) (Haanen et al., 2022; Schneider et al., 2021; Thompson, 2023)

Abbreviation: irAE- immune related adverse event; IBD- inflammatory bowel disease; IO- immunotherapy; iv- intravenous; LFT- liver function test; AE- adverse event.

Arbour et al. (2018), investigated the impact of baseline use of corticosteroids on immunotherapy efficacy in patients with NSCLC. 90 patients (14%) received corticosteroids at baseline, mainly due to dyspnea, fatigue, and brain metastases (Arbour et al., 2018). It was suggested that baseline use of corticosteroids was associated with worse outcome in patients with NSCLC (Arbour et al., 2018). But we should have in mind the reason for corticosteroid use in these patients, so it might be that the disease itself was the reason for the worse outcome (Arbour et al., 2018).

Two other studies also reported worse outcomes in patients with NSCLC who received corticosteroids and who were treated with immunotherapy (Fucà et al., 2019; Scott and Pennell, 2018).

However, in the study conducted by Fucà et al. (2019), the early use of corticosteroids was associated with ECOG PS ≥ 2, presence of brain metastases, and presence of more than 2 metastatic sites.

In the other study the reasons for corticosteroid use were the presence of brain metastases, COPD or other respiratory diseases, disease-related pain, fatigue, and anorexia (Scott and Pennell, 2018).

Thus, in all studies, corticosteroids were used to treat conditions that themselves had worse prognoses.

A meta-analysis also showed that patients who were treated with immunotherapy and used corticosteroids had worse progression free survival (PFS) and overall survival (OS) (Zhang et al., 2021). But the reasons for corticosteroid use in the majority of patients were brain metastases and cancer-related symptoms (Zhang et al., 2021).

Other meta-analysis showed the same results regarding outcomes in patients with NSCLC treated with immunotherapy which used corticosteroids for cancer-related symptoms (Li et al., 2023). But when corticosteroids were used for irAEs management or for non-cancer related indications, it had no impact on outcomes (Li et al., 2023).

Similar results were found in a study conducted by Skribek et al. (2021). Only corticosteroid use for cancer-related symptoms was an independent predictor of worse OS, but when applied due to irAEs did not have an impact on survival (Skribek et al., 2021). Further, they showed that the time of corticosteroid administration did not affect OS (Skribek et al., 2021).

Umehara et al. (2021) found that patients who were treated with nivolumab and to whom corticosteroids were administered before nivolumab initiation had significantly lower PFS and OS compared to patients who did not receive corticosteroids and compared to patients who did receive corticosteroids but after the nivolumab initiation. And there was no difference in PFS and OS in patients who did not receive corticosteroids compared to patients who did receive it, but after the nivolumab initiation (Umehara et al., 2021). Thus, they concluded that it was safe to administer corticosteroids after immunotherapy initiation (Umehara et al., 2021).

However, Huang et al. (2024) suggested in their study that early short-course corticosteroid administration after each immunotherapy application may reduce the rate of irAEs that lead to immunotherapy discontinuation.

In a large retrospective study, more than 2000 patients with melanoma, NSCLC and urothelial cancer who were treated with immunotherapy were included in the analysis (Drakaki et al., 2020). They investigated whether the baseline corticosteroid use had an impact on clinical outcomes (Drakaki et al., 2020). They found that those patients had a 23%–47% increased risk of death compared to patients who did not use corticosteroids (Drakaki et al., 2020). But those patients who used baseline corticosteroids were more likely to have advanced stage of the disease at the diagnosis, distant metastases including liver and brain (which are known to be independent poor prognostic factor), and poorer ECOG PS (Drakaki et al., 2020).

Further, Ricciuti et al. (2019) found that patients who did receive corticosteroids for cancer-related symptoms at the time of immunotherapy initiation had shorter PFS and OS compared to patients who did not use it. But patients who did receive corticosteroids at the time of immunotherapy initiation but for cancer-unrelated symptoms did not have shorter PFS and OS compared to those patients who did not receive corticosteroids (Ricciuti et al., 2019).

Sorial et al. (2021) investigated whether corticosteroid use before the initiation of chemo-immunotherapy had an impact on outcomes in patients with lung cancer. The results showed that corticosteroid use was not associated with worse outcomes (Sorial et al., 2021).

Corticosteroids are inevitable in the treatment of pruritus, acneiform rash, and maculo-papular rash caused by targeted therapy (Wu and Lacouture, 2018). In the treatment of grade 1 or 2, it is recommended to use topical steroid creams, but in the treatment of grade 3 or 4, in addition to topical steroids, oral or iv corticosteroids are also indicated (Wu and Lacouture, 2018). Further, in the treatment of dry skin grade 3, steroids are also indicated (Wu and Lacouture, 2018).

## 4 Palliation of symptoms

### 4.1 Anorexia and cachexia

Cachexia represents a syndrome that could be a part of numerous chronic or terminal diseases, including lung cancer (Fearon et al., 2011; Peixoto da Silva et al., 2020).

It is characterized by systemic inflammation, progressive weight loss, and depletion of adipose tissue and skeletal muscle, and it cannot be reversed by conventional nutritional support (Peixoto da Silva et al., 2020; Pandey et al., 2024). The presence of cancer cachexia is not associated with the tumor size, and the incidence varies among different tumors (Peixoto da Silva et al., 2020; Pandey et al., 2024). It is estimated that cachexia is present in about half of patients with lung cancer. Further, it is shown that chemotherapy and radiotherapy can contribute to the cancer cachexia (Peixoto da Silva et al., 2020; Pandey et al., 2024). Platinum based therapies are associated with weight loss, fatigue and inflammation in cancer patients (Peixoto da Silva et al., 2020; Pandey et al., 2024).

Pin et al. (2019) demonstrated that cancer-induced and chemotherapy-induced cachexia share some metabolic abnormalities.

The underlying mechanism is inhibition of the synthesis/release of pro-inflammatory cytokines and increasing the levels of NPY, with the rapid effects on appetite (Peixoto da Silva et al., 2020).

Anorexia is frequently observed in patients with cancer, and it is associated with limited food intake, worse disease outcomes, increased morbidity and mortality and reduced quality of life (Peixoto da Silva et al., 2020). It could be present in half of the cancer patients at the moment of diagnosis (Peixoto da Silva et al., 2020). There are some factors that can contribute to the development of cachexia such as the administration of chemotherapy, depression, constipation, emesis, dysphagia, stomatitis/mucositis, altered taste, pain, and others (Peixoto da Silva et al., 2020).

Corticosteroids are widely used in treatment of cachexia and anorexia.

Results of a study conducted by Matsuo et al. (2017), suggested that response to corticosteroids may be predicted by Palliative Performance Scale, drowsiness and baseline symptom intensity. Patients with locally advanced and metastatic disease were included (180 of them) who had an anorexia intensity score of 4 or more (Numerical Rating Scale from 0 to 10) (Matsuo et al., 2017). They wanted to identify factors that could predict a two-point reduction or more on an NRS on day 3. 55% of patients had a response to corticosteroids (Matsuo et al., 2017). Palliative Performance Scale> 40 and absence of drowsiness were identified as factors that could predict response and also ECOG PS 0-3, absence of diabetes mellitus, absence of peripheral edema, presence of lung metastasis, absence of peritoneal metastasis, baseline anorexia NRS>6, presence of pain and presence of constipation (Matsuo et al., 2017).

### 4.2 Fatigue

Fatigue is the most common symptom reported by patients from the diagnosis to the end of life (Fabi et al., 2020; Bower, 2014; Horneber et al., 2012). It is defined as a distressing, persistent, subjective sense of physical, emotional and/or cognitive tiredness or exhaustion related to cancer or cancer treatment that is not proportional to recent physical activity and that interferes with usual functioning (Fabi et al., 2020; Bower, 2014; Horneber et al., 2012).

It affects two-thirds of patients, and up to 40% of patients reported fatigue at the moment of diagnosis, and the majority of them reported to have fatigue during the specific cancer treatment (Fabi et al., 2020). 80%–90% had fatigue during chemotherapy and/or radiotherapy, among them 17%–21% during chemotherapy alone and 33%–53% during the association of chemotherapy and radiotherapy (Fabi et al., 2020). New therapies such as molecular therapy and immunotherapy can also cause fatigue (Fabi et al., 2020). It was estimated that fatigue is present in up to 37% of patients treated with immunotherapy and in up to 71% when combined with chemotherapy, monoclonal antibodies, antiangiogenic agents and targeted therapies (Fabi et al., 2020). Further in patients who are treated with immunotherapy fatigue can also be present as a symptom of endocrinopathies (Fabi et al., 2020). Table 4 shows the frequency of cancer related fatigue.

**TABLE 4:** Frequency of cancer related fatigue.

Time of occurrence during disease course	No of patients (%)
At the moment of diagnosis	Up to 40%
Chemotherapy alone	17%–21%
During association of chemotherapy and radiotherapy	33%–53%
Immunotherapy	Up to 37%
Immunotherapy combined with chemotherapy, monoclonal antibodies, antiangiogenic agents and targeted therapies	Up to 71%

Paulsen et al. (2014) investigated whether the addition of methylprednisolone to opioids had an impact on analgesia at day 7 in patients with advanced cancer. Secondary outcomes were analgesic consumption, fatigue and appetite loss (Paulsen et al., 2014). After 7 days there was no difference in pain intensity between the group of patients who received methylprednisolone and the group of patients who did not (Paulsen et al., 2014). Also the difference was not observed regarding analgesic consumption but a significant difference was found in fatigue, appetite loss and patient satisfaction in favor of the group of patients who received methylprednisolone (Paulsen et al., 2014). No difference in adverse events was observed (Paulsen et al., 2014).

In a study conducted by Yennurajalingam et al. (2013), it was investigated whether the fatigue could be reduced with dexamethasone. Patients treated with dexamethasone had reduced fatigue compared to patients who received placebo (Yennurajalingam et al., 2013).

In another study conducted by Matsuo et al. (2016), patients with locally advanced and metastatic disease were included (179 of them) who also had a fatigue intensity score of 4 or more (Numerical Rating Scale from 0 to 10) (Matsuo et al., 2016). They wanted to identify factors that could predict a two-point reduction or more on an NRS on day 3. 48% of patients had a response to treatment with corticosteroids (Matsuo et al., 2016). They concluded that response to corticosteroids may be predicted by baseline symptom intensity, performance status, drowsiness, and severity of fluid retention symptoms (Matsuo et al., 2016).

Sandford et al. (2023), conducted a meta-analysis, and they included three studies that compared corticosteroids to placebo, and one study compared dexamethasone to modafinil. The dose and the duration of the treatment could not be evaluated due to insufficient data in the included studies (Sandford et al., 2023). This meta-analysis could not confirm the benefit of corticosteroid use for treatment of cancer related fatigue at 1 week of the intervention (Sandford et al., 2023). In a study that compared dexamethasone to modafinil, there was an improvement in both groups after 2 weeks, but without a significant difference between groups (Sandford et al., 2023).

Thus, NCCN and ESMO (Fabi et al., 2020) guidelines recommend corticosteroids in the treatment of cancer related fatigue.

### 4.3 Dyspnea

Dyspnea is a common symptom in patients with cancer, with the prevalence between 20% and 70%, especially among patients with thoracic malignancies (Mori et al., 2023). The etiology varies and it could be related to the cancer itself (primary lung cancer and lung metastases, malignant pleural effusion, major airway obstruction, lymphangitis carcinomatosis, superior vena cava syndrome, and respiratory muscle fatigue due to cancer cachexia), cancer treatment (pneumonitis caused by anti-cancer treatment and post-radiation pneumonitis) and comorbidities (Mori et al., 2023).

A nationwide survey among physicians in Japan was conducted in order to investigate the use of corticosteroids in cancer patients (Suzuki et al., 2019). It was found that the majority of them use corticosteroids to treat dyspnea, but one third of them responded that they used them routinely while others used corticosteroids for specific conditions such as lymphangitic carcinomatosis, SVC syndrome, and major airway obstruction (Suzuki et al., 2019).

In a study conducted by Mori et al. (2023), Mori et al. (2017), Hui et al. (2021), potential predictive factors for response to CS were investigated. They revealed that even a lower dose of systemic corticosteroids of 4 mg of dexamethasone or equivalent led to dyspnea relief on day 3 in about two-thirds of patients (Mori et al., 2023; Mori et al., 2017; Hui et al., 2021). Older age, absence of liver metastases, better performance status, presence of pleuritis carcinomatosis with small pleural effusions, presence of audible wheezes, and severe baseline dyspnea were identified as factors associated with dyspnea relief (Mori et al., 2023; Mori et al., 2017; Hui et al., 2021).

ESMO and ASCO guidelines recommend the use of corticosteroids in the treatment of dyspnea in some situations (Hui et al., 2021; Hui et al., 2016; Hui et al., 2020). The efficacy of CS was investigated in a study that compared oral dexamethasone and placebo (Hui et al., 2021; Hui et al., 2016; Hui et al., 2020). It was concluded that dexamethasone may provide rapid relief from dyspnea (Hui et al., 2021; Hui et al., 2016; Hui et al., 2020).

A large confirmatory trial was conducted and it showed that a high dose of dexamethasone did not improve dyspnea compared to placebo (Hui et al., 2022).

In several studies dyspnea was investigated as a secondary outcome. In a previously mentioned study conducted by Yennurajalingam et al. (2013), whose primary endpoint was fatigue, 4 mg of dexamethasone or placebo was administered twice daily for 14 days. It showed positive results for dyspnea also (Yennurajalingam et al., 2013).

Another study investigated the efficacy of dexamethasone compared to placebo in patients who were undergoing radiation for bone metastases (Chow et al., 2015). They found that dexamethasone improved radiation-induced pain flare, but also significantly improved dyspnea (Chow et al., 2015).

Some case reports and case series reported that corticosteroids may be helpful for central airway obstruction, lymphangitic carcinomatosis, and superior vena cava syndrome (Hui et al., 2021).

A meta-analysis conducted by Haywood et al. (2019) investigated the efficacy of systemic corticosteroids in patients with cancer and dyspnea. The results could not confirm the role of corticosteroids in the treatment of dyspnea (Haywood et al., 2019).

Further, pulmonary treatment-related toxicity could be the reason for the development of dyspnea (Jovanovic et al., 2024). It could be presented as asymptomatic, but also it could be presented as a very serious condition with respiratory compromise which requires urgent therapeutic procedures (Jovanovic et al., 2024). Thus, new therapeutic options such as TKIs and immunotherapy could induce the development of pneumonitis, but also chemotherapy and radiotherapy (Jovanovic et al., 2024). Thus, precaution is necessary especially in patients with pre-existing chronic pulmonary diseases (Jovanovic et al., 2024).

Radiotherapy is an important part of the treatment in patients with lung cancer. But after the radiotherapy of lung tumors, post-radiation pneumonitis is always present. It is diagnosed 3–12 weeks after radiotherapy with the symptoms of low-grade temperature, shortness of breath, nonproductive cough and crackles on the physical exam (Gould Rothberg et al., 2022; Li et al., 2019), and fibrotic changes are seen in the irradiated field (Gould Rothberg et al., 2022; Li et al., 2019). The treatment of post-radiation pneumonitis involves the use of corticosteroids. Li et al. (2019) investigated whether the use of corticosteroids had an influence on recurrence and survival outcomes in patients with early stage of NSCLC who were treated with stereotactic ablative radiotherapy (SABR). It was included 912 patients in the study (Li et al., 2019). 87 of them received corticosteroids with SABR, mainly due to other conditions and diseases (Li et al., 2019). The results showed that patients who were treated with corticosteroids had poorer OS, but corticosteroid administration was not associated with shorter PFS (Li et al., 2019).

### 4.4 Nausea and vomiting

Nausea and vomiting is a common symptom in cancer patients (Vayne-Bossert et al., 2017). It could be related to applied cancer treatment-chemotherapy, immunotherapy, targeted therapy, radiotherapy, surgery, but also it could be unrelated to cancer treatment (Vayne-Bossert et al., 2017). Approximately 70% of patients can suffer from nausea and it can have a great impact on quality of life (Vayne-Bossert et al., 2017).

One study evaluated the efficacy of corticosteroids in the treatment of nausea and vomiting not related to cancer treatment (Vayne-Bossert et al., 2017). Three studies were included in a final analysis (Vayne-Bossert et al., 2017). The two of them compared dexamethasone with placebo and the third study compared some additional interventions in various combinations, including metoclopramide, chlorpromazine, tropisetron, and dexamethasone (Vayne-Bossert et al., 2017). Two studies had the duration of 8 days and the third study did not have available data at the same time point (Vayne-Bossert et al., 2017). After 8 days, there was less nausea in a group of patients treated with dexamethasone compared to placebo, but the difference was not statistically significant (Vayne-Bossert et al., 2017). Regarding the side effects of corticosteroids there was no difference between groups (Vayne-Bossert et al., 2017). Thus, authors could not conclude that corticosteroids are effective in this setting (Vayne-Bossert et al., 2017).

In the prevention of chemotherapy-induced nausea and vomiting, corticosteroids are also used. When the cancer treatment contains highly emetogenic agents, besides NK1 receptor antagonists, 5- HT3 receptor antagonists and olanzapine, corticosteroids are part of antiemetic therapy (Yao et al., 2023; Gupta et al., 2021; Aapro et al., 2022). For patients treated with moderate emetic risk agents, 5- HT3 receptor antagonists and dexamethasone are the therapies of choice (Yao et al., 2023; Gupta et al., 2021; Aapro et al., 2022). And in patients who receive low emetic-risk agents, 5- HT3 receptor antagonists or dexamethasone should be offered (Yao et al., 2023; Gupta et al., 2021; Aapro et al., 2022). Table 5.

**TABLE 5:** Management of CINV *(Modified from 2023 MASCC and ESMO guideline update)*.

Emetic risk group by applied therapies	Acute phase	Delayed phase
High (AC and no AC)	5-HT3- RA + DEX + NK1-RA + OLZ	5-HT3- RA + DEX + NK1-RA + olanzapine DEX + OLZ 10 mg on days 2–4 If aprepitan 125 mg is used on day 1, then aprepitant 80 mg on days 2 and 3
Carboplatin	5-HT3- RA + DEX + NK1- RA	1- day DEX If aprepitan 125 mg is used on day 1, then aprepitant 80 mg on days 2 and 3
Moderate (other than carboplatin)	5-HT3- RA + DEX	1- day DEX
Low	5- HT3- RA or DEX or DOP	No routine prophylaxis
Minimal	No routine prophylaxis	No routine prophylaxis

NK1-RA: neurokinin 1 receptor antagonist such as APREPITANT or FOSAPREPITANT or ROLAPITANT or NEPA (combination of netupitant/fosnetupitant and palonosetron); AC: antracycline/cyclophosamide; 5-HT3: 5-hydroxitriptamine or serotonin; DEX: dexamethasone (any corticosteroid); OLZ: olanzapine; DOP: dopamine receptor antagonist

• OLZ 10 mg is effective and tolerable as a rescue antiemetic for breaktrough in patients who did not receive olanzapine as part of the primary antiemetic prophylaxis

Further, corticosteroids are also used for radiotherapy-induced nausea and vomiting. Table 6.

**TABLE 6:** Radiotherapy and chemo-radiotherapy emetic risk relevant in lung cancer and management *(Modified from 2023 MASCC and ESMO guideline update)*.

Radiotherapy	Antiemetic
Total body RT- high risk	Prophylaxis with 5-HT3- RA + DEX
Brain- low risk	Rescue with DEX
Thoracic RT- low emetic risk	Rescue with DEX, a dopamine/RA or 5- HT3- RA
Concomitant RT and weekly cisplatin 40mg/m2	Acute: day 1 before cisplatin prophylaxis with 5-HT3- RA + DEX and APR/fosAPR Delayed: DEX day 2–4
Concomitant chemiradiotherapy	The antiemetic prophylaxis is according to the chemotherapy- related antiemetic guidelines of the corresponding risk category, unless the risk of emesis is higher with RT than chemotherapy

RT: radiotherapy; 5-HT3: 5-hydroxitriptamine or serotonin; DEX: dexamethasone (any corticosteroid); APR/fosAPR: aprepitant/fosaprepitant

### 4.5 Spinal cord compression

Spinal cord compression is a very serious complication of lung cancer, because it can lead to permanent neurological impairment, including incontinence, loss of sensory and motoric functions, and finally to paraplegia (Yao et al., 2023). It was found that around 20% of patients with lung cancer may present with spinal cord compression as the first symptom of the disease (Yao et al., 2023). It develops by hematogenous spread into vertebral-body mass, by pathological fractures which lead to epidural compression or by direct tumor extension into the vertebral column (Yao et al., 2023; Gould Rothberg et al., 2022). It is advised to start corticosteroid therapy as soon as possible, ideally within 12 h from diagnosis in order to reduce the edema (Yao et al., 2023; Gould Rothberg et al., 2022). The dosing of dexamethasone usually starts with 10 mg once, followed by 4 mg every 6 h (Yao et al., 2023; Gould Rothberg et al., 2022). Surgical intervention should be considered, and if surgery is not an option, radiation must be considered for symptom palliation (Yao et al., 2023; Gould Rothberg et al., 2022).

### 4.6 Pain

The role of corticosteroids in pain control is to reduce inflammation which is caused by tissue injury (Leppert and Buss, 2012). It is also suggested that pathological electrical activity of damaged neurons is also decreased (Leppert and Buss, 2012). The anti-inflammatory effect is a result of their ability to inhibit the expression of collagenase (the key enzyme involved in tissue degeneration during inflammatory mechanisms), to reduce pro-inflammatory cytokines, and to stimulate the synthesis of lipocortin (blocks the production of eicosanoids) (Leppert and Buss, 2012). The steroids also have anti-swelling effects, which may help in the reduction of peritumoral edema and thus in pain reduction in patients with brain metastases and spinal cord compression (Leppert and Buss, 2012).

The most commonly prescribed corticosteroid for pain management is dexamethasone, because it has minimal effect on fluid retention and less mineralocorticoid effect than other steroids (Leppert and Buss, 2012).

Neuropathic pain could be characterized as numbness, hot or cold prickling, or electric sensations, with or without muscle weakness (Yao et al., 2023; Gould Rothberg et al., 2022). It might be due to the tumor pressing the nerves or more often due to some chemotherapy agents, such as platinum based treatments or taxanes (Yao et al., 2023; Gould Rothberg et al., 2022). This type of pain may not have a response to opioids, and the drugs that are usually prescribed are gabapentin, pregabalin, duloxetine and corticosteroids (Yao et al., 2023; Gould Rothberg et al., 2022), but many patients have symptom relief after several weeks (Gould Rothberg et al., 2022) or do not have pain relief despite the optimal therapy (Yao et al., 2023).

Paulsen et al. (2013), investigated the efficacy of corticosteroids in the cancer pain management. They included 4 studies with 667 patients in the final analysis (Paulsen et al., 2013). Among the included patients, one of the most predominant primary tumors was lung cancer (Paulsen et al., 2013). One study showed significant pain reduction and lower use of analgesics (Paulsen et al., 2013). In another study, the addition of corticosteroids had no effect on pain intensity (Paulsen et al., 2013). And in two others the data about analgesic consumption were not adequately reported (Paulsen et al., 2013).

Bruera et al. (1985), conducted a study to evaluate the effect of corticosteroids (methylprednisolone 32 mg) compared to placebo in 40 patients with advanced cancer. They evaluated its effects on pain, psychiatric status, appetite, nutritional status, daily activity, and performance (Bruera et al., 1985). After 14 days all patients were given corticosteroids for 20 days (Bruera et al., 1985). 70% of patients had bone, visceral or neuropathic pain (Bruera et al., 1985). Visual analogue scale score and analgesic use were lower in a group of patients treated with corticosteroids in all types of pain, but the benefit disappeared after 33 days in one third of patients (Bruera et al., 1985). Regarding other symptoms they were all improved: appetite in 77% of patients, daily activity in 68% of patients and depression in 71% of patients (Bruera et al., 1985).

A Swedish study evaluated the efficacy of corticosteroids in patients with advanced cancer and specific and non-specific indications, pain was one of them (Lundström and Fürst, 2006). About one third of patients had a very good effect and almost half of patients had some effect in pain treatment (Lundström and Fürst, 2006).

## 5 Conclusion

Corticosteroids are widely used in different clinical situations in patients with lung cancer, but without clear evidence for their efficacy. In the majority of cases clinical improvement was made and it was shown that corticosteroids are safe when used for a short time.
